# Post-marketing safety evaluation of lurbinectedin: a pharmacovigilance analysis based on the FAERS database

**DOI:** 10.3389/fphar.2024.1368763

**Published:** 2024-03-14

**Authors:** Zhao Li, Changying Guo, Xingfei Liu, Zhengzhou Qiu, Ruilin Zhang

**Affiliations:** ^1^ Department of Thoracic Surgery, Jiangxi Cancer Hospital, The Second Affiliated Hospital of Nanchang Medical College, Jiangxi Cancer Institute, Nanchang, China; ^2^ Jiangxi Medical College, Nanchang University, NanChang, China

**Keywords:** lurbinectedin, small cell lung cancer, FAERS, pharmacovigilance, adverse events

## Abstract

**Background:** On 15 June 2020, the United States Food and Drug Administration (FDA) approved lurbinectedin for treating adult patients with metastatic small-cell lung cancer whose disease has progressed despite prior platinum-based chemotherapy. Following its market approval, safety data on lurbinectedin in large populations is currently lacking. Therefore, this study aims to evaluate adverse events (AEs) associated with lurbinectedin using the FDA’s Adverse Event Reporting System (FAERS)database.

**Methods:** Data concerning lurbinectedin from the FAERS database were extracted for the period from June 2020 to September 2023. Four disproportionality analysis algorithms were utilized to assess potential AEs linked to lurbinectedin: reporting odds ratio (ROR), proportional reporting ratio, disproportionate multi-item gamma Poisson shrinker, and Bayesian confidence propagation neural network. These algorithms were applied to quantify signals of lurbinectedin-related AEs.

**Result:** A total of 5,801,535 AE reports were retrieved from the FAERS database, with 511 related to lurbinectedin. These lurbinectedin-induced AEs were observed in 23 system organ classes (SOCs). After simultaneously applying the four algorithms, 47 lurbinectedin-induced AE signals were detected in 23 SOCs. At the SOC level, blood and lymphatic system disorders (ROR, 6.70; 95% confidence interval [CI]: 5.47–8.22) were the only SOC that met all four algorithms. Lurbinectedin’s most frequent adverse event was death (ROR: 6.11%, 95% CI: 4.86–7.68), while extravasation exhibited the strongest signal intensity in the ROR algorithm (ROR: 326.37%, 95% CI: 191.66–555.75). Notably, we identified a novel signals: tumor lysis syndrome (ROR: 63.22%, 95% CI: 33.87–117.99). The mean time of onset of AEs was 66 days, the median time of onset was 25 days (interquartile range: 8–64 days), and most AEs occurred within the first month of lurbinectedin treatment.

**Conclusion:** Our study provided a comprehensive evaluation of lurbinectedin’s safety profile in the post-marketing setting. In addition to the adverse events consistent with the existing clinical trials and labeling information, we have also identified an unreported signal related to tumor lysis syndrome. This finding will better guide the clinical practice of lurbinectedin and provide valuable evidence for future research.

## 1 Introduction

Lung cancer represents a significant burden of disease. The most recent cancer statistics indicate that it is the primary cause of cancer-related mortality globally, ranking first for both men and women. Additionally, its overall incidence ranks second among both genders (Siegel et al., 2022). Small cell lung cancer (SCLC) constitutes −15% of all lung cancer cases and is linked to a notably high mortality rate, especially in males (Kalemkerian et al., 2013; Torre et al., 2016).SCLC can be categorized into limited-stage (LS-SCLC) and extensive-stage (ES-SCLC), with the latter representing −65% of new cases (Kalemkerian et al., 2013). SCLC is an aggressive high-grade neuroendocrine malignancy with a bleak prognosis. It is highly aggressive and associated with poor outcomes (Torre et al., 2016). Following an untreated SCLC diagnosis, the median overall survival (OS) is 2–4 months. However, with treatment, patients with LS-SCLC exhibit a median OS of 16–24 months, in contrast to 6–12 months for those with ES-SCLC (PDQ Adult Treatment Editorial Board, 2002). For SCLC, the preferred initial standard chemotherapy involves combining etoposide or irinotecan with platinum. Following this treatment, SCLC typically experiences improved disease control; nevertheless, most patients encounter recurrence shortly thereafter, often with additional metastatic sites (Ito et al., 2017).

Until 2020, limited options existed for second-line chemotherapy regimens for SCLC, with topotecan being the only approved drug. However, topotecan’s substantial hematologic toxicity restricts its suitability for patients with SCLC. Additionally, the OS for patients treated with topotecan was only 26 weeks (O’Brien et al., 2006). With the United States Food and Drug Administration’s (FDA’s) approval of lurbinectedin in June 2020 for adult patients with metastatic SCLC whose disease has progressed after or during platinum-based chemotherapy, a new era has begun for the second-line treatment of patients with SCLC (FDA, 2020). Lurbinectedin, a derivative of herein, is an alkylating agent and an inhibitor of RNA polymerase II (Pol II). It can covalently bind to the grooves on the DNA double helix structure, forming adducts, which produce DNA double-strand breaks, thereby disrupting DNA-protein interactions and RNA transcription (Leal et al., 2010; Santamaría Nuñez et al., 2016).

Exciting progress has been observed in a high-profile clinical trial of lurbinectedin in metastatic SCLC. Notably, in the single-arm, open-label, phase 2 basket trial (Study B-005; NCT02454972), lurbinectedin received FDA approval for marketing. In this study, 105 patients were treated with 3.2 mg/m^2^ of lurbinectedin every 21 days until disease progression, and an overall objective response rate (ORR) of 35% (95% confidence interval [CI]: 26.6%, 45.3%) was found (Trigo et al., 2020, p. 2). On the FDA label for lurbinectedin, the documented adverse reactions primarily stem from assorted clinical trials. The most prevalent adverse reactions (with an incidence equal to or greater than 20%) include hematologic toxicity, fatigue, increased creatinine, hepatotoxicity, increased glucose levels, nausea, loss of appetite, constipation, dyspnea, vomiting, cough, decreased magnesium, diarrhea, musculoskeletal pain, decreased albumin, etc .,(FDA, 2020). The adverse reactions associated with lurbinectedin are derived from clinical trial findings. As the utilization of lurbinectedin in clinical practice expands, there is a growing likelihood of encountering potential adverse reactions that have not been previously reported. Additionally, the constrained number of patients and limited follow-up duration in clinical trials may not entirely assess the toxicity profile of lurbinectedin. To enable a comprehensive post-marketing safety assessment of lurbinectedin, we employed the FDA Adverse Event Reporting System (FAERS) database. This initiative aims to furnish clinicians and pharmacists with a vital point of reference for managing the safety of lurbinectedin.

## 2 Materials and methods

### 2.1 Data sources and study design

We conducted a retrospective pharmacovigilance analysis encompassing all adverse effect reports of lurbinectedin from June 2020 to September 2023, utilizing adverse reaction data sourced from the FAERS database. The FDA established the FAERS database as a publicly accessible repository for compiling global adverse reaction reports, facilitating post-marketing safety monitoring of drugs (Kaland et al., 2022). The database not only includes adverse event (AE) reports submitted to the FDA but also encompasses reports of medication errors and product quality complaints leading to AEs, typically reported by pharmacists, medical professionals, consumers, and others. The FAERS database comprises eight document types: Demographic and Administrative Information, Drug Information (DRUG), Indications for Use, Reporting Source, Start and End Dates of Reported Drugs, Patient Prognosis, as well as Invalidity Reporting, and Adverse Events (Yang et al., 2022). Access to specific data is available on the FDA’s website: https://fis.fda.gov/extensions/FPD-QDE-FAERS/FPD-QDE-FAERS.html. This study does not necessitate informed consent or ethical approval since the FAERS data are publicly available, and patient information in adverse reaction reports is anonymized.

### 2.2 Data extraction

To mitigate the impact of duplicate reports in the FAERS database stemming from submissions by various sources, we implemented a strategy recommended by the FDA. Specifically, we utilized the CASEID report identifier to select the most recent FDA_DT (date the report was received by the FDA) and the highest primary drug (Shu et al., 2022).

In the FAERS database, each report is coded using the Medical Dictionary of Regulatory Activities, with hierarchical terminology systems such as system organ class (SOC), high-level group term, high-level term, preferred term (PT), and lowest-level term (Mascolo et al., 2021). For our pharmacovigilance analysis related to lurbinectedin, we focused on the SOC and PT levels to facilitate the identification of potential safety signals. To capture all relevant reports, we searched for the DRUG file using the terms lurbinectedin, its former name PM-01183, and its brand name Zepzelca. We applied the primary suspect criterion as one of the required screening criteria.

### 2.3 Data mining and statistical analysis

Disproportionate analysis stands as the widely accepted method for signal monitoring in pharmacovigilance analysis (Hu et al., 2020). This approach compares the proportion of specific adverse reactions associated with single or multiple drugs to the proportion of adverse reactions reported for the same drug across the database, as depicted in Table 1. Common algorithms for disproportionate analysis encompass report odds ratios (ROR), proportional adverse drug reaction reporting ratio (PRR), multi-item gamma-Poisson shrinker (MGPS), and Bayesian confidence propagation neural network (Bate et al., 1998; Evans et al., 2001; Szarfman et al., 2002; van Puijenbroek et al., 2002). The formulas and standards for these algorithms are provided in Table 2. To mitigate deviations in signal detection outcomes and ensure accurate detection, we assert that an AE signal can only be identified when all four algorithm conditions are simultaneously satisfied. All data processing and statistical analyses were performed using R v4.3.1.

**TABLE 1:** Four-grid table for signal detection.

Type of drug	*N* of target adverse reaction reports	*N* of other adverse reaction reports	Total
Target drug	*a*	*b*	*a*+*b*
Other drugs	*c*	*d*	*c* + *d*
Total	*a*+*c*	*b* + *d*	*N* = *a*+*b* + *c* + *d*

**Table 2:** Overview of the main algorithms used for signal detection.

Algorithm	Publicity	Standard for generating signals
ROR	$ROR=\frac{a/c}{b/d}$	Lower 95% CI>1, *N*≥2
PRR	$PRR=\frac{a/\left(a+b\right)}{c/\left(c+d\right)}$	χ^2^≥4, PRR≥2, *N*≥3
	$x^{2}=\sum \left[{\left(O-E\right)}^{2}/E\right];O=a,E=\left(a+b\right)\left(a+c\right)/\left(a+b+c+d\right)$	
MGPS	$EBGM=\frac{a\left(a+b+c+d\right)}{\left(a+b\right)\left(a+c\right)}$	Lower 95% CI>2
BCPNN	$IC=\log_{2}\frac{a\left(a+b+c+d\right)}{\left(a+b\right)\left(a+c\right)}$	Lower 95% CI>0, *N*>0

ROR: Reporting odds ratio; PRR: Proportional reporting ratio; BCPNN: Bayesian confidence propagation neural network; MGPS: Multi-item gamma Poisson shrinker; EBGM: Empirical Bayesian ensemble mean; IC: Information component; CI: Confidence interval.

## 3 Result

### 3.1 Descriptive analysis

**FIGURE 1:**
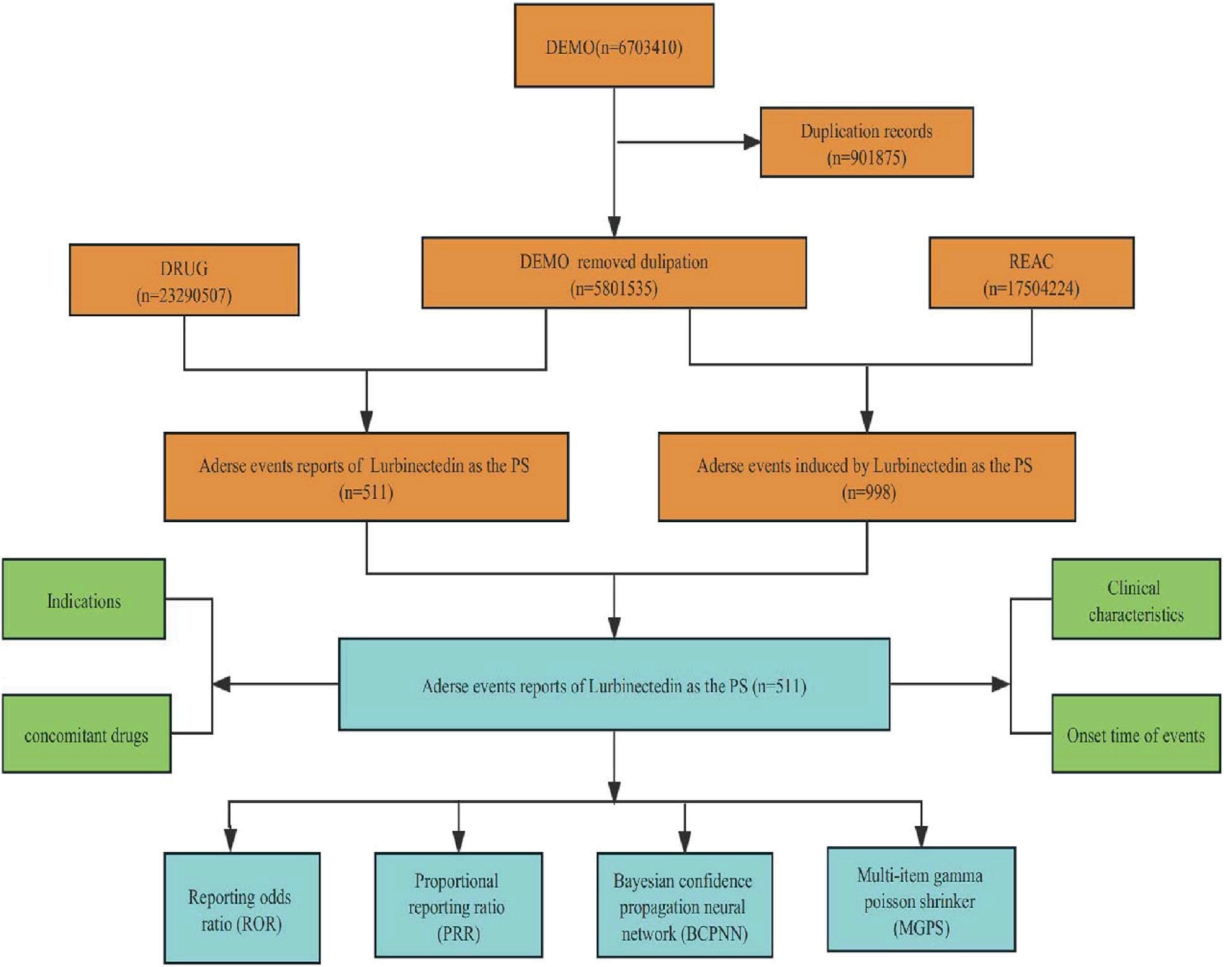
Flowchart of the screening process for lurbinectedin-related adverse events.

Throughout the surveillance period spanning from 2020 to 2023 Q3, the FAERS database yielded a total of 5,801,535 reports. The detailed data mining process and results are presented in Figure 1. After eliminating duplicate reports, we identified a total of 511 reports suspected to be related to lurbinectedin. Furthermore, we summarized the clinical features, indications for lurbinectedin, concomitant medications, and timing of adverse reactions associated with these 511 reports.

Table 2 showcases the clinical characteristics of events associated with lurbinectedin. Among the 511 reported cases of AEs induced by lurbinectedin, males (37.6%) experienced a higher impact compared to females (30.9%). Analyzing age groups, the occurrence of events was similar between the 18 to 64 and 65 to 85 brackets, accounting for 19.6% and 22.3%, respectively.

Since the FDA approved lurbinectedin in 2020, the lowest number of cases occurred in that year (21, 4.2%), while the highest was recorded in 2021 (209, 40.9%). Moreover, the number of cases in 2022 was lower than those documented in the first three quarters of 2023 (126 [24.6%] vs. 155 [30.3%]). In terms of reporting countries, the United States reported the highest number of AEs (48.7%), followed by Canada (17.2%), France (7.6%), and others.

Concerning reporting sources, pharmacists and physicians submitted 319 AE reports, constituting 62.5% of the total. Consumers submitted an additional 104 reports, representing 20.4% of the total. Furthermore, there were 145 reported cases of death, making up 19.9% of the total reports, and 137 cases resulting in hospitalization, accounting for 18.8% of the total. Other serious AEs, including instances of disability and life-threatening situations, were also reported.

Table 3 outlines the top 5 concomitant drugs and their respective indications for lurbinectedin. The primary indications are SCLC, prophylaxis, metastatic SCLC, ES-SCLC, and hypertension (after removing unknown indications). The most frequently used medications with lurbinectedin are dexamethasone and ondansetron, followed by irinotecan, atezolizumab, and carboplatin.

**TABLE 3:** Top 5 indications and concomitant drugs of lurbinectedin from FAERS database.

Characteristics	Variable	Case number
Indications	Small cell lung cancer (SCLC)	220
	Prophylaxis	75
	Metastatic SCLC	56
	Extensive stage SCLC	51
	Hypertension	32
Concomitant drugs	Dexamethasone	63
	Ondansetron	52
	Irinotecan	47
	Atezolizumab	47
	Carboplatin	44

### 3.2 Disproportionality analysis

#### 3.2.1 SOC level

**FIGURE 2:**
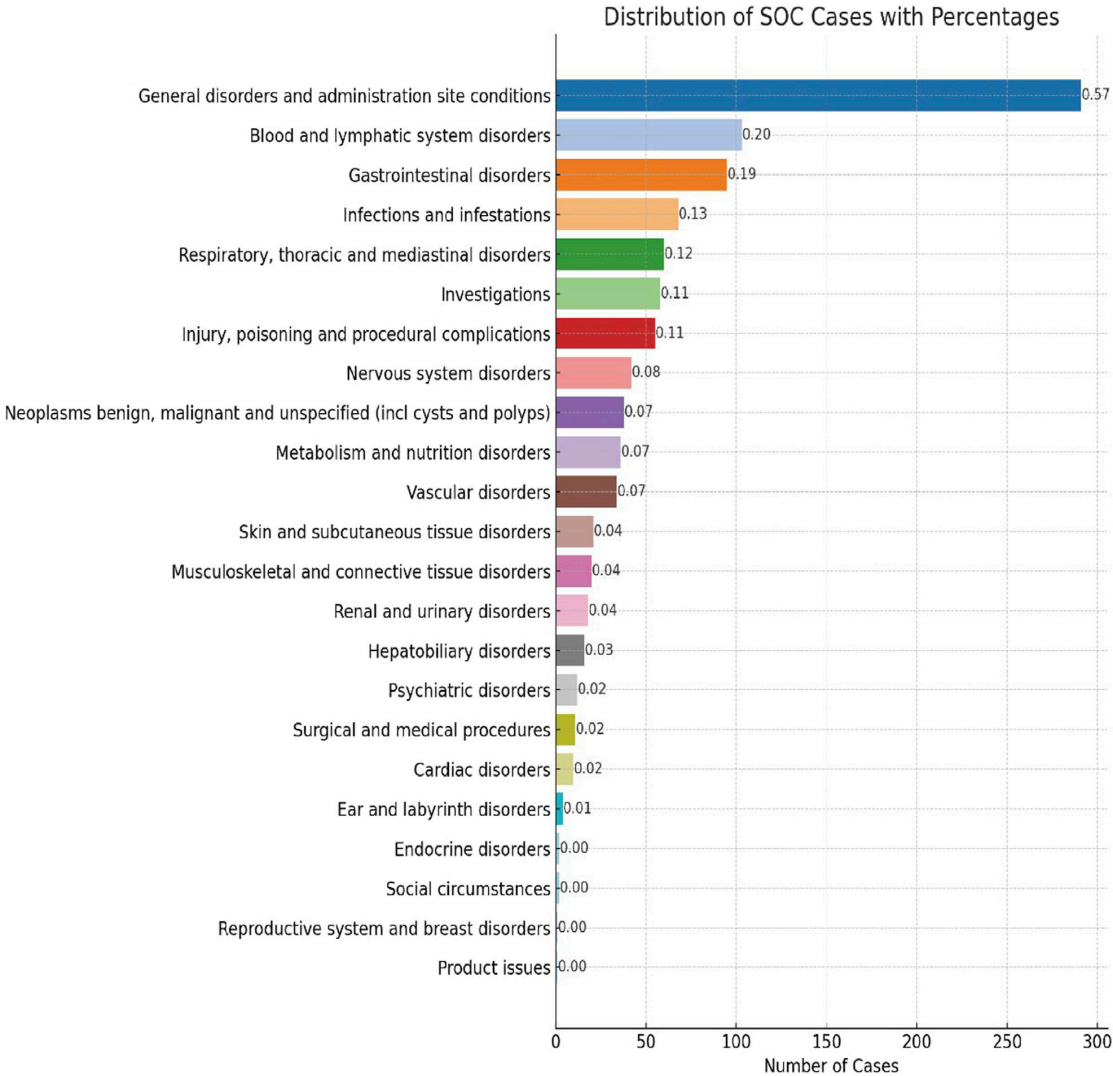
Proportion of system organ classes (SOCs) reported in lurbinectedin-related adverse events (AEs).

AE reports associated with lurbinectedin documented a total of 23 SOCs. The most frequently reported SOCs included general disorders and administration site conditions (SOC: 10018065), blood and lymphatic system disorders (SOC: 10005329), and gastrointestinal disorders (SOC: 10017947). Additionally, there were notable occurrences of AE events in infections and infestations (SOC: 10021881) and respiratory, thoracic, and mediastinal disorders (SOC: 10038738), with 68 and 60 cases, respectively. Among these, blood and lymphatic system disorders are the sole SOCs that simultaneously meet all four criteria (ROR: 6.70%, 95% CI: 5.47–8.22). Figure 2 visually represents the distribution of AE report frequencies across the various SOCs.

Furthermore, Table 4 presents the ROR and its 95% CI, PRR and chi-square, empirical Bayesian geometric mean (EBGM) and the lower bound of its 95% CI EBGM_05_, along with the lower bound of the 95% CI for the information component IC_025_. These values are calculated using four different algorithms.

**TABLE 4:** Signal strength of lurbinectedin-related reports in the FAERS database at the system organ class (SOC) level.

System organ class (SOC)	Cases	ROR (95% CI)	PRR (χ^2^)	EBGM (EGBM_05_)	IC (IC_025_)
General disorders and administration site conditions	291	1.90 (1.66–2.18)	1.64 (89.17)	1.64 (1.43)	0.72 (−0.95)
Blood and lymphatic system disorders*	103	6.70 (5.47–8.22)	6.11 (448.09)	6.11 (4.99)	2.61 (0.94)
Gastrointestinal disorders	95	1.25 (1.01–1.54)	1.22 (4.27)	1.23 (0.99)	0.29 (−1.37)
Infections and infestations	68	1.24 (0.97–1.58)	1.22 (2.88)	1.22 (0.95)	0.29 (−1.38)
Respiratory, thoracic, and mediastinal disorders	60	1.37 (1.05–1.77)	1.34 (5.56)	1.34 (1.04)	0.43 (−1.24)
Investigations	58	0.99 (0.76–1.29)	0.99 (0.01)	0.99 (0.76)	−0.02 (−1.69)
Injury, poisoning, and procedural complications	55	0.42 (0.32–0.55)	0.45 (41.12)	0.45 (0.35)	−1.14 (−2.81)
Nervous system disorders	42	0.57 (0.42–0.77)	0.58 (13.40)	0.58 (0.43)	−0.77 (−2.44)
Neoplasms benign, malignant, and unspecified (incl. cysts and polyps)	38	0.85 (0.62–1.18)	0.86 (0.92)	0.86 (0.62)	−0.22 (−1.88)
Metabolism and nutrition disorders	36	1.95 (1.40–2.72)	1.91 (15.99)	1.91 (1.37)	0.94 (−0.73)
Vascular disorders	34	1.87 (1.33–2.64)	1.84 (13.35)	1.84 (1.31)	0.88 (−0.78)
Skin and subcutaneous tissue disorders	21	0.37 (0.24–0.58)	0.39 (21.48)	0.39 (0.25)	−1.36 (−3.03)
Musculoskeletal and connective tissue disorders	20	0.38 (0.24–0.58)	0.39 (20.34)	0.39 (0.25)	−1.36 (−3.03)
Renal and urinary disorders	18	0.95 (0.59–1.52)	0.95 (0.04)	0.95 (0.59)	−0.06 (−1.73)
Hepatobiliary disorders	16	2.02 (1.23–3.31)	2.00 (8.09)	2.00 (1.22)	1.00 (−0.66)
Psychiatric disorders	12	0.21 (0.12–0.37)	0.22 (35.55)	0.22 (0.12)	−2.19 (−3.86)
Surgical and medical procedures	11	0.78 (0.43–1.40)	0.78 (0.71)	0.78 (0.43)	−0.36 (−2.03)
Cardiac disorders	10	0.51 (0.27–0.95)	0.51 (4.72)	0.51 (0.27)	−0.96 (−2.63)
Ear and labyrinth disorders	4	0.99 (0.37–2.65)	0.99 (0.00)	0.99 (0.37)	−0.01 (−1.68)
Endocrine disorders	2	0.78 (0.19–3.12)	0.78 (0.13)	0.78 (0.19)	−0.35 (−2.02)
Social circumstances	2	0.42 (0.10–1.67)	0.42 (1.62)	0.42 (0.10)	−1.25 (−2.92)
Reproductive system and breast disorders	1	0.16 (0.02–1.16)	0.16 (4.26)	0.16 (0.02)	−2.60 (−4.26)
Product issues	1	0.05 (0.00–0.38)	0.05 (16.42)	0.05 (0.00)	−4.17 (−5.84)

*Represents the simultaneous fulfillment of four algorithms; CI: confidence interval; EGBM_05_: lower limit of the 95% two-sided CI, for empirical Bayes geometric mean; IC_025_: lower limit of the 95% two-sided CI, for the information component.

#### 3.2.2 PT level

**FIGURE 3:**
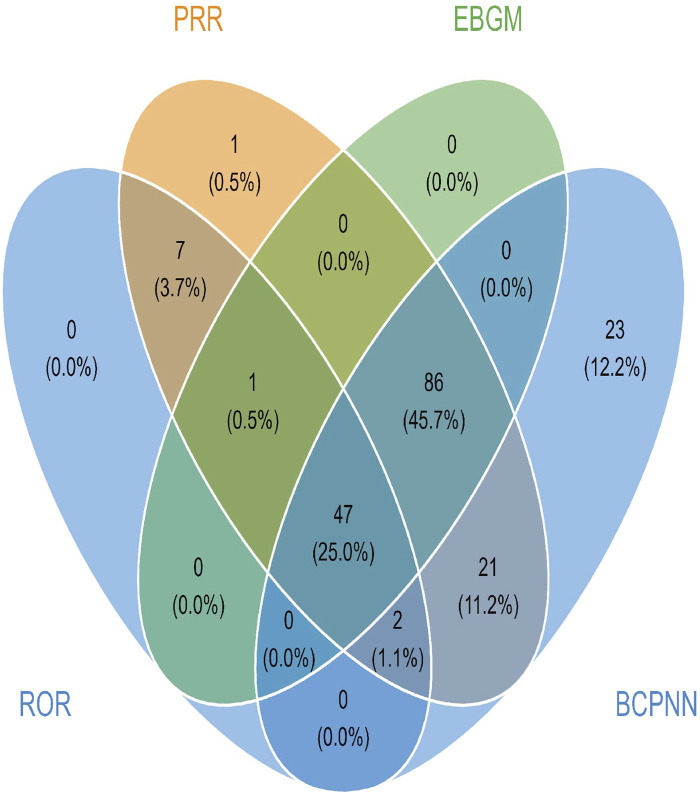
Venn diagram of preferred term (PT) signals meeting the criteria of four algorithms.

**FIGURE 4:**
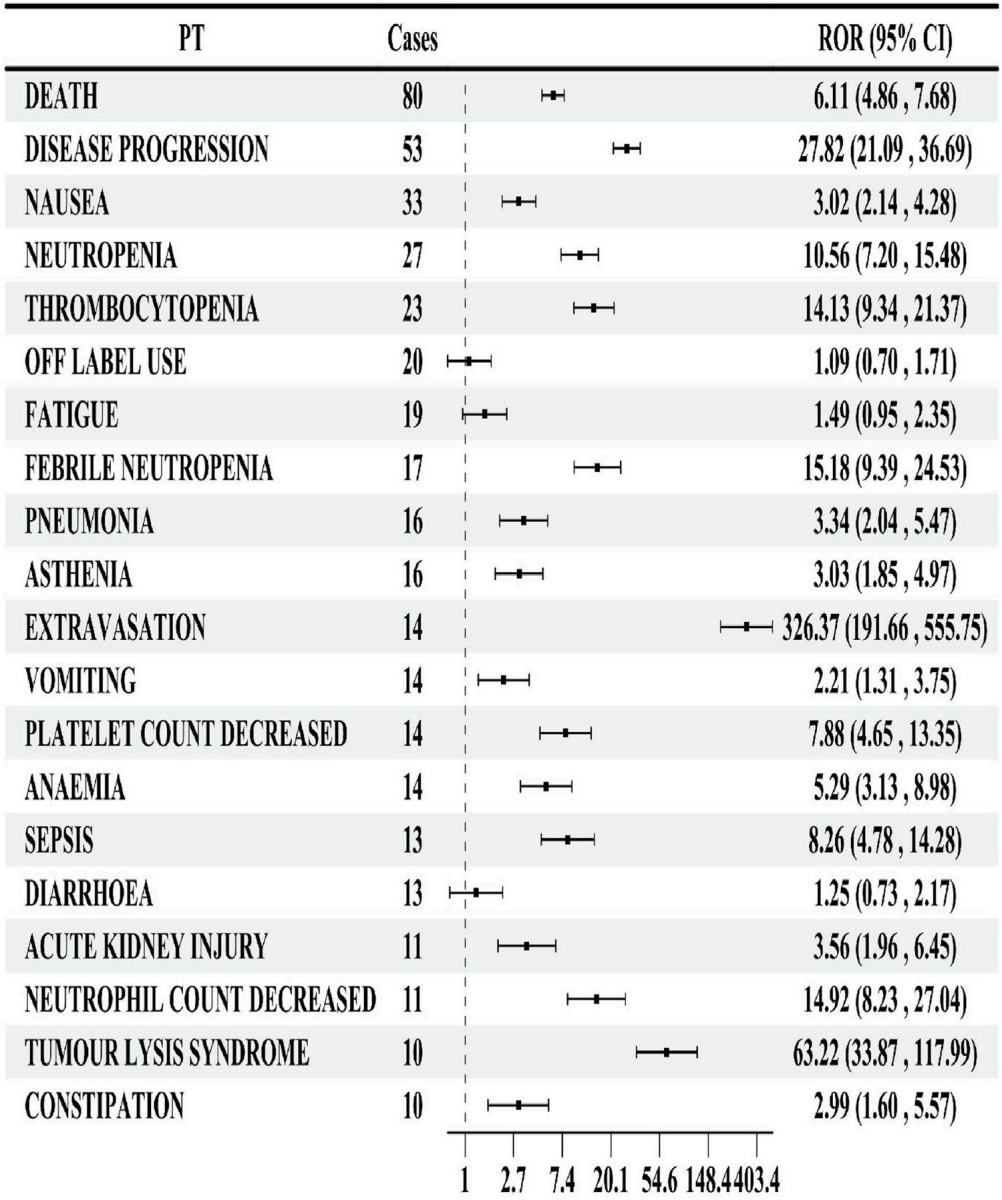
Forest map of the reporting odds ratio (ROR) of the top 20 preferred terms (PTs) by number of reports.

In lurbinectedin-related AEs, a total of 310 PTs were reported. Employing four distinct algorithms, we identified 47 PT signals. Figure 3 visually represents a Venn diagram illustrating the PT signals compliant with standards, identified by all PTs after the application of the four algorithms. Table 5 provides specific reports of PTs with AEs exceeding 10, along with the outcomes of each algorithm. A total of 13 PTs satisfy all four algorithms simultaneously: death (PT: 10011906), anemia (PT: 10002034), disease progression (PT: 10061818), extravasation (PT: 10015866), neutropenia (PT: 10016288), thrombocytopenia (PT: 10043554), febrile neutropenia (PT: 10016288), tumor lysis syndrome (PT: 10045170), platelet count decreased (PT: 10051608), neutrophil count decreased (PT: 10029366), pneumonia (PT: 10035664), sepsis (PT: 10040047), and acute kidney injury (AKI; PT: 10069339).Lurbinectedin’s most frequent adverse event was death (ROR: 6.11%, 95% CI: 4.86–7.68), while extravasation exhibited the strongest signal intensity in the ROR algorithm (ROR: 326.37%, 95% CI: 191.66–555.75). Notably, we identified two novel signals: tumor lysis syndrome (ROR: 63.22%, 95% CI: 33.87–117.99) and pneumonia (ROR: 3.34%, 95% CI: 2.04–5.47). Additionally, Figure 4 displays the forest plot of the ROR values for the top 20 events. For more detailed results of PT signal intensity, refer to Supplementary Table S1.

**TABLE 5:** Signal strength of lurbinectedin-related reports in the FAERS database at the preferred term (PT) level.

System organ class (SOC)	PTs	*N*	ROR (95% CI)	PRR (χ^2^)	EBGM (EGBM_05_)	IC (IC_025_)
General disorders and administration site conditions	Death*	80	6.11 (4.86–7.68)	5.70 (314.42)	5.70 (4.71)	2.51 (0.84)
	Disease progression*	53	27.82 (21.09–36.70)	26.40 (1,295.81)	26.36 (20.91)	4.72 (3.05)
	Fatigue	19	1.49 (0.95–2.35)	1.49 (3.05)	1.49 (1.02)	0.57 (−1.10)
	Asthenia	16	3.03 (1.85–4.97)	3.00 (21.47)	3.00 (1.99)	1.58 (−0.08)
	Extravasation*	14	326.37 (191.66–555.75)	321.80 (4,396.82)	316.02 (202.44)	8.30 (6.63)
Gastrointestinal disorders	Nausea	33	3.02 (2.14–4.28)	2.96 (43.19)	2.96 (2.21)	1.56 (−0.10)
	Vomiting	14	2.21 (1.31–3.75)	2.20 (9.18)	2.20 (1.41)	1.13 (−0.53)
	Diarrhoea	13	1.25 (0.73–2.17)	1.25 (0.66)	1.25 (0.79)	0.32 (−1.35)
	Constipation	10	2.99 (1.60–5.57)	2.97 (13.09)	2.97 (1.76)	1.57 (−0.10)
Injury, poisoning and procedural complications	Off label use	20	1.10 (0.70–1.71)	1.09 (0.16)	1.09 (0.75)	0.13 (−1.54)
Blood and lymphatic system disorders	Neutropenia*	27	10.56 (7.20–15.48)	10.30 (227.22)	10.30 (7.48)	3.36 (1.70)
	Thrombocytopenia*	23	14.13 (9.34–21.37)	13.83 (273.93)	13.82 (9.77)	3.79 (2.12)
	Febrile neutropenia*	17	15.18 (9.40–24.53)	14.94 (221.19)	14.93 (9.99)	3.90 (2.23)
	Anaemia*	14	5.30 (3.13–8.98)	5.24 (48.12)	5.24 (3.37)	2.39 (0.72)
Metabolism and nutrition disorders	Tumour lysis syndrome*	10	63.22 (33.87–117.99)	62.59 (604.02)	62.37 (37.00)	5.96 (4.29)
Investigations	Platelet count decreased*	14	7.88 (4.65–13.36)	7.78 (82.88)	7.78 (5.00)	2.96 (1.29)
	Neutrophil count decreased*	11	14.92 (8.23–27.04)	14.77 (141.17)	14.76 (8.97)	3.88 (2.21)
Infections and infestations	Pneumonia*	16	3.34 (2.04–5.47)	3.30 (25.75)	3.30 (2.18)	1.72 (0.05)
	Sepsis*	13	8.26 (4.78–14.28)	8.17 (81.84)	8.16 (5.16)	3.03 (1.36)
Renal and urinary disorders	Acute kidney injury*	11	3.56 (1.96–6.45)	3.53 (20.01)	3.53 (2.15)	1.82 (0.15)

*Represents the simultaneous fulfillment of four algorithms; CI: confidence interval; EGBM_05_: lower limit of the 95% two-sided CI, for empirical Bayes geometric mean; IC_025_: lower limit of the 95% two-sided CI, for the information component.

### 3.3 Time-to-onset analysis

**FIGURE 5:**
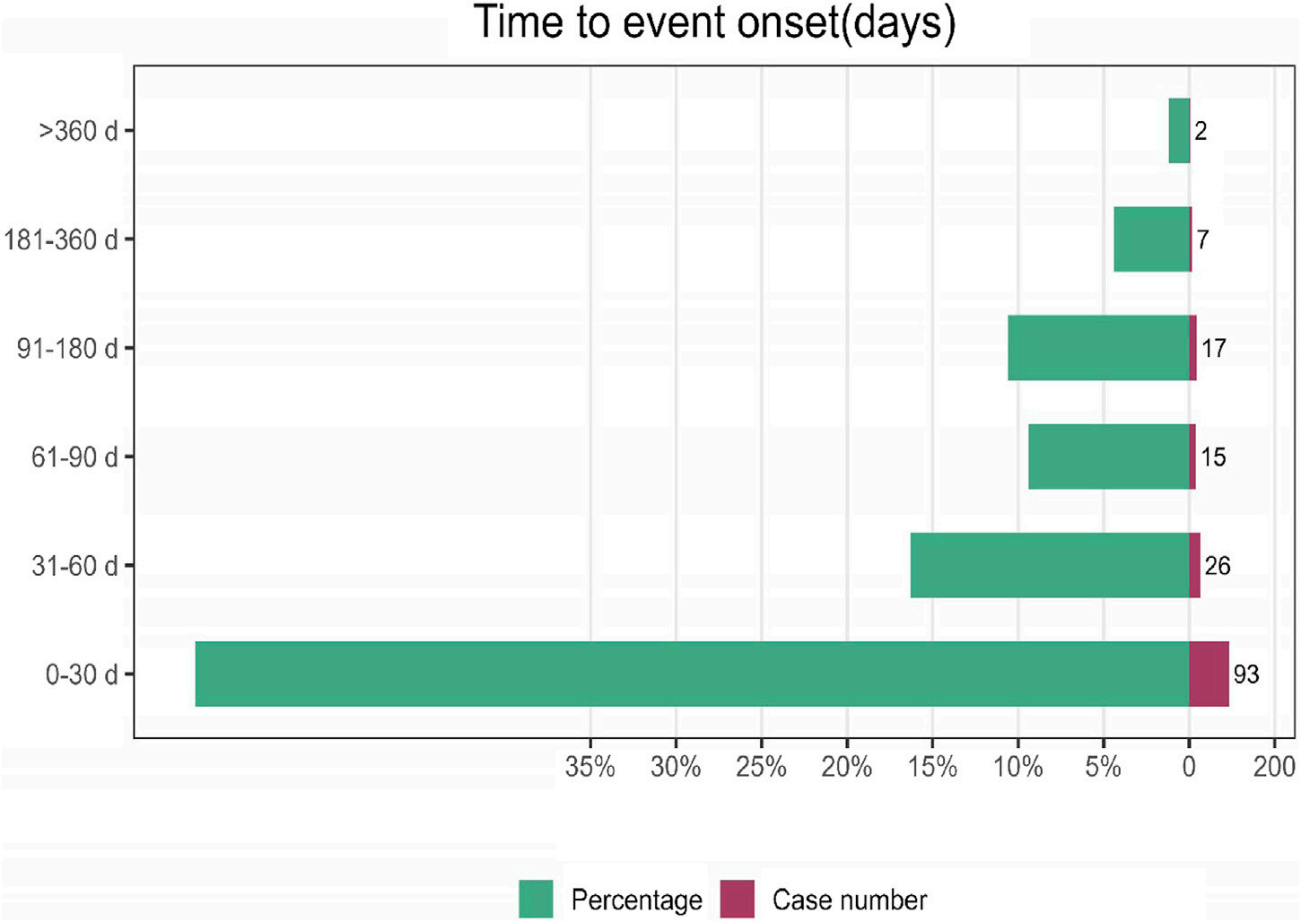
Onset time distribution of lurbinectedin-related adverse events (AEs).

Among −160 AE reports, information on the time to onset was available. The average onset time was 66 days, with a median of 25 days (interquartile range [IQR]: 8–64 days). Figure 5 visually represents the specific onset times and their proportions in the total reports. The highest incidence of AEs, accounting for 58% of all reports, was observed within the first 30 days. In contrast, AEs occurring after 6 months of treatment were the least frequent, constituting only 5% of reports. The number of AEs within 61–90 days of treatment was comparable to the number of AEs occurring 91–180 days after treatment.

### 3.4 Analysis of signals

#### 3.4.1 Analysis of established signals

We reviewed lurbinectedin reports for the indications of Metastatic SCLC and Extensive stage SCLC and performed a disproportionate analysis to focus on major signals. This aimed to determine differences in adverse events (AE) attributable to lurbinectedin compared to other treatment regimens for patients with metastatic small cell lung cancer, such as topotecan. In the comparative analysis with topotecan and other drugs, we observed that death (ROR: 15.19%, 95% CI:9.59-24.06) and disease progression (ROR:51.81%, 95% CI:27.91-96.19) remained the most common AEs associated with lurbinectedin. In addition, major signals including thrombocytopenia (ROR:18.25%, 95% CI:6.74-49.40), platelet count decreased (ROR:8.82%, 95% CI6.95-50.96), neutrophil count decreased (ROR:38.88%, 95% CI:12.37-122.22), and extravasation (ROR:227.52%, 95% CI:56.25-920.29) were concurrently detected by all four algorithms. Furthermore, the new signal of tumor lysis syndrome (ROR; 120.20%, 95% CI:29.72-486.09), previously identified, also met all four algorithms simultaneously. More detailed results of the disproportionate analysis at the PT level are provided in Supplementary Table S2.

#### 3.4.2 Analysis of new signals

We conducted a confounding factor analysis on the newly confirmed signals of tumor lysis syndrome and pneumonia to validate their accuracy. Tumor lysis syndrome is clinically associated with acute kidney failure. To ascertain if the reports of acute kidney failure in our adverse event (AE) reports are linked to tumor lysis syndrome, we analyzed them in conjunction. Supplementary Table S3 presents the clinical characteristics of acute kidney injury (AKI), tumor lysis syndrome, and pneumonia reports, comprising a total of 35 reports with an equal distribution between male and female patients (13 reports each) and the majority of reports (22, 62.8%) originating from the United States. Additionally, Supplementary Table S4 displays the combined report numbers and concomitant medication situations for these three signals. The combined reports of AKI and tumor lysis syndrome were zero, indicating that AKIs are attributed to lurbinectedin rather than tumor lysis syndrome. Furthermore, the combined reports of pneumonia and tumor lysis syndrome were found to be 2. Notably, concomitant medications for all three signals were absent, suggesting that these AEs were solely caused by lurbinectedin.

Given the high infection status of potential lung cancer patients for pneumonia, we performed disproportionate analyses of AE reports for lurbinectedin’s primary indications (small cell lung cancer, metastatic SCLC, and extensive stage SCLC) separately to ascertain if pneumonia was induced by lurbinectedin. The disproportionate analyses of AE reports for small cell lung cancer and metastatic SCLC are provided in Supplementary Tables S2, S5, respectively. Following these analyses, we determined that pneumonia did not simultaneously satisfy the four algorithms, thereby leading us to exclude it from the new signal of lurbinectedin.

## 4 Discussion

SCLC represents a significant public health threat owing to its significant associated morbidity and mortality rate. The management of metastatic SCLC remains a clinical challenge, particularly following the failure of initial platinum-based therapy. Currently, only topotecan is approved for use in such cases. Lurbinectedin, a synthetic anticancer agent derived from marine sources, selectively inhibits oncogenic transcription and exhibits a more favorable safety profile compared to topotecan, as indicated by the results of current data.

In this study, lurbinectedin demonstrated no treatment-related deaths (in contrast to 7.9%–11.2% for topotecan), a lower rate of discontinuation due to drug toxicity (2% vs. 27%), and reduced incidences of grade 3–4 anemia, neutropenia, and thrombocytopenia (9% vs. 26.1%–30.5%, 46% vs. 53.8%–78.4%, and 7% vs. 45.5%–54.3%, respectively). Furthermore, lurbinectedin exhibited significantly higher ORR, progression-free survival, and OS compared to topotecan (35.2% vs. 24.3%, 3.5 months vs. 3.1 months, and 9.1 months vs. 5.8 months, respectively) (Baize et al., 2020; Trigo et al., 2020; Evans et al., 2015).

Despite these promising results, the safety profile of lurbinectedin remains incompletely understood due to the limitations of current studies, which are confined to clinical trials and case reports with small sample sizes and short follow-up periods. Moreover, case reports cannot definitively establish a causal relationship between lurbinectedin and AEs. This study aims to offer a more comprehensive characterization of lurbinectedin-associated AEs by analyzing data from the FAERS database, providing the most accurate and comprehensive description of lurbinectedin-associated AEs to date.

From June 2020 to September 2023, we extracted 511 reports of lurbinectedin as the primary suspect drug from the FAERS database, excluding duplicate entries. Among these cases, males slightly outnumbered females (192 vs. 158). This observation aligns with the epidemiological characteristics of SCLC, which exhibits a higher prevalence among males. However, it is noteworthy that the proportion of female SCLC patients has been steadily increasing over the past 5 decades. This trend may be associated with changing tobacco consumption patterns. Nevertheless, the incidence of SCLC remains lower in females compared to males, which is consistent with the gender distribution in our study (Rudin et al., 2021). The age group of 65–85 years was prominently represented, aligning with the typical onset age of SCLC. Adverse reaction reports to lurbinectedin peaked in 2021, slightly decreased in 2022, and showed a gradual rise in 2023, possibly influenced by promising clinical trial results involving lurbinectedin. Nearly half of the reports originated from the United States, likely due to lurbinectedin’s initial introduction there, while only four relevant reports came from China, reflecting the drug’s absence of approval in the country until now.The FAERS database contains drug safety reports from various countries worldwide. Several clinical trials are currently underway to evaluate the efficacy of lurbinectedin in treating extensive-stage small cell lung cancer (ES-SCLC). Two notable trials include the NCT04638491 trial from China and the NCT05285033 trial from France (Desai et al., 2023). The inclusion of these trials helps explain the presence of reports from China and France in Table 6.

**TABLE 6:** Clinical characteristics of patients with adverse events caused by lurbinectedin.

Characteristics	Variable	*N* (%)
Overall		511 (100%)
Gender	Male	192 (37.6%)
	Female	158 (30.9%)
	Unknown	161 (31.5%)
Age (years)	18–64	100 (19.6%)
	65–85	114 (22.3%)
	>85	3 (0.6%)
	Unknown	294 (57.5%)
Report year	2020	21 (4.2%)
	2021	209 (40.9%)
	2022	126 (24.6%)
	2023Q3	155 (30.3%)
Report country	United States	249 (48.7%)
	Canada	88 (17.2%)
	France	39 (7.6%)
	China	4 (0.8%)
	Others	131 (25.6%)
Reporter’s occupation	Consumer	104 (20.4%)
	Pharmacist	123 (24.1%)
	Physician	196 (38.4%)
	Unknown	88 (17.2%)
Outcome	Death	145 (19.9%)
	Disability	2 (0.3%)
	Hospitalization	137 (18.8%)
	Life-threatening	12 (1.6%)
	Other serious medical event	305 (41.9%)
	Unknown	128 (17.6%)

Q3: The first three quarters.

Notably, a majority of adverse reaction reports for lurbinectedin were submitted by medical professionals, predominantly pharmacists and physicians, accounting for 319 cases or 62.5% of all reports. The outcome section documented 296 cases of serious outcomes, including death, disability requiring hospitalization, and life-threatening conditions. Regarding indications, SCLC constituted the largest proportion with 220 reports, consistent with the FDA’s approval of lurbinectedin for treating SCLC that recurs after first-line platinum-based therapy. It is noteworthy that there were 32 reports indicating the potential use of lurbinectedin in the treatment of hypertension. This may be attributed to its ability to inhibit the production of inflammatory cytokines, such as IL-6, IL-8, and MCP-1, which are known to contribute to rapid wasting of the body and depletion of muscle and heart tissues (Belgiovine et al., 2017; Argilés et al., 2019). After correlating pro-inflammatory cytokines with lurbinectedin, studies have suggested that in mouse models, lurbinectedin could potentially enhance myocardial function. However, it did not achieve statistical significance in these experiments. Yet, the effectiveness of lurbinectedin in mitigating myocardial damage in mice has been acknowledged. This finding offers a theoretical rationale for considering the use of lurbinectedin in preventing myocardial damage in hypertensive patients (Aquila et al., 2020). Dexamethasone and ondansetron were the most frequently reported concomitant drugs, in line with lurbinectedin’s FDA label recommendations to prevent vomiting in patients receiving lurbinectedin. Irinotecan, carboplatin, and atezolizumab were also observed as concomitant drugs, but their occurrences were relatively low (47, 44, and 47 reports, respectively), given lurbinectedin’s typical use as monotherapy for metastatic SCLC, as indicated by the FDA.

The ATLANTIS study demonstrated the feasibility of combining lurbinectedin with chemotherapy drugs in patients with recurrent SCLC, showing a median OS of 8.6 months (95% CI: 7.1–9.4) in the combination group compared to 7.6 months (95% CI: 6.6–8.2) in the control group receiving topotecan alone. Grade 3 or greater hematologic AEs were less frequent in the combination group, with incidences of anemia, neutropenia, and thrombocytopenia being notably lower compared to the control group (19% [57 of 302 patients] vs. 38% [110 of 288 patients], 37% [112 patients] vs. 69% [200 patients], and 14% [42 patients] vs. 31% [90 patients], respectively) (Aix et al., 2023). This phase III trial provides clinical evidence supporting lurbinectedin’s use in combination with other drugs for the treatment of recurrent SCLC. Anticipating further clinical trials, the combination of chemotherapy drugs with other anti-tumor agents has emerged as a promising strategy for treating malignant tumors, demonstrating excellent and effective treatment outcomes. This may also explain why lurbinectedin is recommended as a monotherapy and why it has been utilized in combination with drugs like irinotecan and cisplatin. The analysis of the reports suggests that lurbinectedin might be administered following the failure of chemotherapy drugs, or it could be employed in combination pending further clinical trial confirmation.

Among the 511 lurbinectedin-related AEs, we identified 23 SOCs and 310 PTs. At the PT level, a total of 47 signals were identified, but our discussion focused on PTs with events greater than or equal to 10, as signals with smaller sample sizes are considered less reliable. General disorders and administration site conditions were the most common SOCs, with the most frequent PTs being death, disease progression, fatigue, asthenia, and extravasation. Extravasation, death, and disease progression were identified as new signals by four algorithms, indicating a potential association with lurbinectedin. However, extravasation and fatigue/asthenia are already documented in the FDA label for lurbinectedin. Therefore, new signals generated by death and disease progression may be attributed to the characteristics of metastatic SCLC itself. The only SOC consistently meeting the criteria of all four algorithms was blood and lymphatic system disorders, with a ROR of 6.70 (95% CI: 5.47–8.22), a PRR of 6.11 (χ^2^ = 448.09), an EBGM_05_ of 6.11 (4.99), and an IC_025_ of 2.61 (0.94). This finding aligns with hematologic toxicity, the most common adverse reaction associated with lurbinectedin. PTs representing blood toxicity, including neutropenia, thrombocytopenia, febrile neutropenia, anemia, platelet count decreased, and neutrophil count decreased, met the criteria of all four signal detection algorithms.

Extravasation had the highest ROR (95% CI) among all AEs associated with lurbinectedin, with a value of 326.37 (191.66–555.75). Consistent with the label, preventing extravasation during clinical use is emphasized to avoid tissue necrosis. Of note, to minimize the risk of extravasation, the use of a central venous catheter for the administration of lurbinectedin is recommended.Sepsis was identified as a potential signal, and new signals not previously recorded in the label include tumor lysis syndrome (ROR: 63.22%, 95% CI: 33.87–117.99), pneumonia (ROR: 3.34%, 95% CI: 2.04–5.47). Pneumonia, although reported in less than 10% of patients in previous phase 2 trials, may be a potentially common adverse effect of lurbinectedin. Tumor lysis syndrome has been mentioned in one case report, suggesting cytotoxicity of lurbinectedin may be an important consideration for clinical management (Kaland et al., 2022). We performed additional analysis on both the established and newly discovered signals. To facilitate a better comparison of the major adverse events (AEs) associated with lurbinectedin and similar drugs for the treatment of metastatic small cell lung cancer, such as topotecan, we restricted the specified indications to Metastatic SCLC and Extensive stage SCLC. In our comparative analysis, death (ROR: 15.19%, 95% CI: 9.59-24.06) and disease progression (ROR: 51.81%, 95% CI: 27.91-96.19) were persistently the most common AEs linked to lurbinectedin. Furthermore, notable signals, including thrombocytopenia (ROR: 18.25%, 95% CI: 6.74-49.40), reduced platelet count (ROR: 8.82%, 95% CI: 6.95-50.96), decreased neutrophil count (ROR: 38.88%, 95% CI: 12.37-122.22), and extravasation (ROR: 227.52%, 95% CI: 56.25-920.29), were consistently identified by all four algorithms. Moreover, the previously identified new signal of tumor lysis syndrome (ROR: 120.20%, 95% CI: 29.72-486.09) concurrently satisfied all four algorithms. To mitigate confounding factors associated with the new signal, we scrutinized the reports of acute kidney injury (AKI) suspected to be caused by tumor lysis syndrome. The findings indicated that AKI is an independent AE from tumor lysis syndrome and is not affected by concomitant medications in potential new signal reports. For confirming the causation of pneumonia by lurbinectedin rather than solely by the heightened infection status of small cell lung cancer patients, we separately analyzed the main indications for lurbinectedin. The results revealed that pneumonia did not simultaneously meet the criteria of all four algorithms in the disproportional analysis for each primary indication, rendering it unconfirmed as a new signal. Therefore, we only acknowledge tumor lysis syndrome as the new signal for lurbinectedin.

A total of 160 reports of time to onset were recorded, with a mean duration of onset of 66 days and a median duration of onset of 25 days (IQR: 8–64 days). AEs were most frequent within 30 days of treatment initiation, accounting for 58% of all reports (*n* = 93). Conversely, AEs occurring after 6 months of treatment were the least common, comprising only 5% of reports. The number of events with onset after one and 2 months of treatment was 26 and 15, respectively, while only two reports of adverse reactions occurred after 1 year. Given the limitations of our real-world data report, particularly the lack of a large sample size, we are currently unable to definitively determine the long-term AEs associated with lurbinectedin. Additionally, the highly invasive nature and high mortality rate of SCLC may influence our assessment of the time of onset of AEs potentially caused by lurbinectedin. Nevertheless, it is imperative that clinicians pay close attention to lurbinectedin in clinical practice and take appropriate preventive measures for relevant AEs.

The mechanism of action of lurbinectedin involves several key processes. First, it inhibits the transcriptional process of tumor cells by binding to CG-rich sequences, predominantly around the promoters of protein-coding genes. Second, lurbinectedin irreversibly arrests extended RNA Pol II on DNA templates, subsequently degrading them through the ubiquitin/proteasome mechanism. Third, it generates DNA fragments, ultimately leading to apoptosis. This inhibition of RNA Pol II phosphorylation not only prevents its degradation but also hinders DNA repair, thus confirming lurbinectedin’s anti-tumor activity at the molecular level (Santamaría Nuñez et al., 2016).

Recent research has demonstrated additional facets of lurbinectedin’s mechanism. It not only inhibits the active transcription of tumor cells and DNA repair mechanisms but also reduces the number of blood vessels and macrophages in tumor tissues by decreasing circulating monocytes. This alteration in the tumor microenvironment contributes to effective anti-tumor activity. Moreover, lurbinectedin induces caspase-8-dependent apoptosis of human-purified monocytes *in vitro*. It significantly inhibits inflammatory and growth factors such as VEGF, CCL2, and CXCL8 at low doses, diminiss202020hing the adhesion and migration ability of monocytes (Belgiovine et al., 2017).

Based on previous research, the administration of lurbinectedin may result in a decrease in monocyte adhesion function and a weakening of white blood cell migration ability. Additionally, lurbinectedin’s inhibitory effect on DNA repair and its cytotoxic properties may impact the body’s bone marrow hematopoietic system, which experiences the most rapid cell turnover. Consequently, this could lead to various adverse events in the blood system, such as decreased neutrophil and platelet levels. The occurrence of medication leakage may be attributed to lurbinectedin’s strong stimulating effect on peripheral vessels, affecting the permeability of the vascular endothelium. Subsequently, the decrease in neutrophils may lead to the development of additional AEs, notably sepsis. Another study suggests that lurbinectedin may exert immunomodulatory effects by stimulating the proliferation and phenotypic transition of anti-tumor immune cell populations. Notably, lurbinectedin specifically enhances the proliferation of CD4^+^ and CD8^+^ T cells in patients with SCLC, as well as the proliferation of NK and NKT cells in patients with SCLC. These findings provide insights into the underlying mechanism for the development of tumor lysis syndrome associated with the novel signal identified (Arnaud et al., 2022; Dumoulin et al., 2022).

Despite its recent approval for marketing, lurbinectedin currently has limited real-world safety data, and there is a shortage of clinical trials providing comprehensive information. Our study represents the first large-scale assessment of post-marketing AEs associated with lurbinectedin, utilizing the FAERS database to uncover potential safety signals. We systematically explored and analyzed common AE signals, such as death, disease progression, asthenia, and cytologic toxicity, along with other noteworthy AE reports.

However, it is essential to acknowledge the limitations of our study. First, the FAERS database is susceptible to underreporting, incomplete, or inaccurate reporting, which may impact the reliability of our findings. Second, we did not account for potential confounding factors, including drug interactions, which could influence AEs. Third, while disproportionality analysis aids in identifying potential signals, it does not establish a definitive causal relationship between AEs and lurbinectedin.

## 5 Conclusion

We conducted an analysis of 5,801,535 reports obtained from the FAERS database spanning June 2020 to September 2023, excluding duplicate AE reports. Among these, 511 AE reports were linked to lurbinectedin. Utilizing non-proportionality analysis, we successfully identified lurbinectedin-related AE signals, delving into details such as the time of onset, indications of AEs, and concurrent drug use. The most prevalent adverse event is death, and hematologic toxicity is frequently reported in adverse events, aligning with the details in the lurbinectedin label.Additionally, our analysis unveiled novel potential signals associated with lurbinectedin, namely tumor lysis syndrome. This pharmacovigilance assessment enhances our understanding of lurbinectedin’s safety profile, offering valuable evidence for future research and informing clinical practice.

## Data Availability

The original contributions presented in the study are included in the article/Supplementary Material, further inquiries can be directed to the corresponding author.
