# Supplementary material for: Post-marketing safety evaluation of lurbinectedin: a pharmacovigilance analysis based on the FAERS database
Source: Front Pharmacol. 2024 Mar 14;15:1368763. doi: 10.3389/fphar.2024.1368763 (PMC10972959; doi:10.3389/fphar.2024.1368763)
Supplement: Supplementary file 1 [file Table1.DOCX]

| Supplementary Table 1: Signal intensity based on the PT level of lurbinectedin in FAERS. (Number of events in the top 100) | | | | | | | | | | |
| --- | --- | --- | --- | --- | --- | --- | --- | --- | --- | --- |
| PT | Cases | ROR | RORL | RORU | PRR | χ^2^ | EBGM | EBGM05 | IC2 | C025 |
| Death | 80 | 6.11 | 4.86 | 7.68 | 5.7 | 314.42 | 5.7 | 4.71 | 2.51 | 0.84 |
| Disease Progression | 53 | 27.82 | 21.09 | 36.7 | 26.4 | 1295.81 | 26.36 | 20.91 | 4.72 | 3.05 |
| Nausea | 33 | 3.02 | 2.14 | 4.28 | 2.96 | 43.19 | 2.96 | 2.21 | 1.56 | -0.1 |
| Neutropenia | 27 | 10.56 | 7.2 | 15.48 | 10.3 | 227.22 | 10.3 | 7.48 | 3.36 | 1.7 |
| Thrombocytopenia | 23 | 14.13 | 9.34 | 21.37 | 13.83 | 273.93 | 13.82 | 9.77 | 3.79 | 2.12 |
| Off Label Use | 20 | 1.10 | 0.7 | 1.71 | 1.09 | 0.16 | 1.09 | 0.75 | 0.13 | -1.54 |
| Fatigue | 19 | 1.49 | 0.95 | 2.35 | 1.49 | 3.05 | 1.49 | 1.02 | 0.57 | -1.1 |
| Febrile Neutropenia | 17 | 15.18 | 9.4 | 24.53 | 14.94 | 221.19 | 14.93 | 9.99 | 3.9 | 2.23 |
| Pneumonia | 16 | 3.34 | 2.04 | 5.47 | 3.3 | 25.75 | 3.3 | 2.18 | 1.72 | 0.05 |
| Asthenia | 16 | 3.03 | 1.85 | 4.97 | 3 | 21.47 | 3 | 1.99 | 1.59 | -0.08 |
| Extravasation | 14 | 326.37 | 191.66 | 555.75 | 321.8 | 4396.82 | 316.02 | 202.44 | 8.3 | 6.63 |
| Vomiting | 14 | 2.21 | 1.31 | 3.75 | 2.2 | 9.18 | 2.2 | 1.41 | 1.13 | -0.53 |
| Platelet Count Decreased | 14 | 7.88 | 4.65 | 13.36 | 7.78 | 82.88 | 7.78 | 5 | 2.96 | 1.29 |
| Anaemia | 14 | 5.3 | 3.13 | 8.98 | 5.24 | 48.12 | 5.24 | 3.37 | 2.39 | 0.72 |
| Sepsis | 13 | 8.26 | 4.78 | 14.28 | 8.17 | 81.84 | 8.16 | 5.16 | 3.03 | 1.36 |
| Diarrhoea | 13 | 1.25 | 0.73 | 2.17 | 1.25 | 0.66 | 1.25 | 0.79 | 0.32 | -1.35 |
| Acute Kidney Injury | 11 | 3.56 | 1.96 | 6.45 | 3.53 | 20.01 | 3.53 | 2.15 | 1.82 | 0.15 |
| Neutrophil Count Decreased | 11 | 14.92 | 8.23 | 27.04 | 14.77 | 141.17 | 14.76 | 8.97 | 3.88 | 2.21 |
| Tumour Lysis Syndrome | 10 | 63.22 | 33.87 | 117.99 | 62.59 | 604.02 | 62.37 | 37 | 5.96 | 4.29 |
| Constipation | 10 | 2.99 | 1.6 | 5.57 | 2.97 | 13.09 | 2.97 | 1.76 | 1.57 | -0.1 |
| Dyspnoea | 9 | 1.07 | 0.56 | 2.07 | 1.07 | 0.05 | 1.07 | 0.62 | 0.1 | -1.57 |
| Respiratory Failure | 9 | 9.48 | 4.92 | 18.27 | 9.4 | 67.59 | 9.4 | 5.42 | 3.23 | 1.56 |
| Phlebitis | 9 | 166.43 | 86.07 | 321.8 | 164.94 | 1452.9 | 163.41 | 94.12 | 7.35 | 5.68 |
| Malignant Neoplasm Progression | 9 | 5.09 | 2.64 | 9.81 | 5.05 | 29.27 | 5.05 | 2.91 | 2.34 | 0.67 |
| Abdominal Pain | 8 | 2.42 | 1.21 | 4.85 | 2.41 | 6.61 | 2.41 | 1.35 | 1.27 | -0.4 |
| Infusion Related Reaction | 8 | 5.89 | 2.94 | 11.81 | 5.85 | 32.2 | 5.85 | 3.27 | 2.55 | 0.88 |
| Infusion Site Extravasation | 7 | 46.54 | 22.11 | 97.98 | 46.22 | 308.94 | 46.1 | 24.73 | 5.53 | 3.86 |
| Pneumonitis | 7 | 15.48 | 7.36 | 32.58 | 15.38 | 94.09 | 15.37 | 8.25 | 3.94 | 2.27 |
| Metastases To Central Nervous System | 7 | 34.08 | 16.19 | 71.73 | 33.85 | 222.76 | 33.79 | 18.13 | 5.08 | 3.41 |
| Decreased Appetite | 7 | 1.93 | 0.92 | 4.05 | 1.92 | 3.1 | 1.92 | 1.03 | 0.94 | -0.73 |
| Injection Site Reaction | 7 | 9.29 | 4.42 | 19.55 | 9.24 | 51.42 | 9.23 | 4.95 | 3.21 | 1.54 |
| Peripheral Swelling | 6 | 1.9 | 0.85 | 4.24 | 1.9 | 2.55 | 1.9 | 0.97 | 0.92 | -0.75 |
| White Blood Cell Count Decreased | 6 | 3.11 | 1.39 | 6.94 | 3.1 | 8.54 | 3.1 | 1.58 | 1.63 | -0.04 |
| Septic Shock | 6 | 9.42 | 4.22 | 21.03 | 9.37 | 44.87 | 9.37 | 4.78 | 3.23 | 1.56 |
| Infusion Site Pain | 6 | 23.56 | 10.55 | 52.59 | 23.42 | 128.64 | 23.39 | 11.95 | 4.55 | 2.88 |
| Myelosuppression | 6 | 8.07 | 3.61 | 18 | 8.02 | 36.9 | 8.02 | 4.1 | 3 | 1.34 |
| Administration Site Extravasation | 6 | 270.06 | 120.3 | 606.26 | 268.44 | 1574.62 | 264.41 | 134.41 | 8.05 | 6.37 |
| Dehydration | 5 | 2.98 | 1.24 | 7.18 | 2.97 | 6.56 | 2.97 | 1.42 | 1.57 | -0.1 |
| Drug Ineffective | 5 | 0.21 | 0.09 | 0.5 | 0.21 | 14.87 | 0.21 | 0.1 | -2.23 | -3.9 |
| Vein Disorder | 5 | 72.41 | 30.02 | 174.67 | 72.06 | 348.94 | 71.76 | 34.35 | 6.17 | 4.49 |
| Infusion Site Swelling | 5 | 36.76 | 15.25 | 88.6 | 36.58 | 172.72 | 36.51 | 17.49 | 5.19 | 3.52 |
| Chest Pain | 5 | 2.23 | 0.93 | 5.38 | 2.23 | 3.39 | 2.23 | 1.07 | 1.16 | -0.51 |
| Dizziness | 5 | 0.73 | 0.3 | 1.76 | 0.73 | 0.5 | 0.73 | 0.35 | -0.45 | -2.12 |
| Small Cell Lung Cancer | 5 | 259.21 | 106.96 | 628.15 | 257.92 | 1261.08 | 254.19 | 121.2 | 7.99 | 6.31 |
| Hyponatraemia | 5 | 6.44 | 2.67 | 15.51 | 6.41 | 22.85 | 6.41 | 3.07 | 2.68 | 1.01 |
| Fall | 5 | 1.02 | 0.42 | 2.46 | 1.02 | 0 | 1.02 | 0.49 | 0.03 | -1.64 |
| Blister | 4 | 4.72 | 1.77 | 12.62 | 4.71 | 11.7 | 4.71 | 2.07 | 2.24 | 0.57 |
| Renal Failure | 4 | 1.93 | 0.72 | 5.16 | 1.93 | 1.79 | 1.93 | 0.85 | 0.95 | -0.72 |
| Pancytopenia | 4 | 5.22 | 1.96 | 13.94 | 5.21 | 13.6 | 5.2 | 2.29 | 2.38 | 0.71 |
| Cellulitis | 4 | 5.49 | 2.06 | 14.67 | 5.47 | 14.64 | 5.47 | 2.41 | 2.45 | 0.78 |
| Erythema | 4 | 1.21 | 0.45 | 3.23 | 1.21 | 0.14 | 1.21 | 0.53 | 0.27 | -1.4 |
| General Physical Health Deterioration | 4 | 2.14 | 0.8 | 5.72 | 2.14 | 2.43 | 2.14 | 0.94 | 1.1 | -0.57 |
| Atrial Fibrillation | 4 | 2.65 | 0.99 | 7.08 | 2.65 | 4.1 | 2.65 | 1.16 | 1.4 | -0.26 |
| Hypoxia | 4 | 7.23 | 2.71 | 19.31 | 7.21 | 21.38 | 7.2 | 3.17 | 2.85 | 1.18 |
| Rhabdomyolysis | 4 | 8.31 | 3.11 | 22.2 | 8.28 | 25.62 | 8.28 | 3.64 | 3.05 | 1.38 |
| Neuropathy Peripheral | 4 | 2.44 | 0.91 | 6.51 | 2.43 | 3.38 | 2.43 | 1.07 | 1.28 | -0.39 |
| Infusion Site Erythema | 4 | 25.96 | 9.72 | 69.35 | 25.86 | 95.46 | 25.82 | 11.35 | 4.69 | 3.02 |
| Paraesthesia | 4 | 1.82 | 0.68 | 4.85 | 1.81 | 1.46 | 1.81 | 0.8 | 0.86 | -0.81 |
| Neoplasm Progression | 4 | 4.67 | 1.75 | 12.46 | 4.65 | 11.48 | 4.65 | 2.05 | 2.22 | 0.55 |
| Colitis | 4 | 6.37 | 2.39 | 17.01 | 6.35 | 18.03 | 6.35 | 2.79 | 2.67 | 1 |
| Respiratory Distress | 3 | 8.61 | 2.77 | 26.74 | 8.58 | 20.1 | 8.58 | 3.32 | 3.1 | 1.43 |
| Transaminases Increased | 3 | 8.84 | 2.84 | 27.46 | 8.81 | 20.78 | 8.81 | 3.41 | 3.14 | 1.47 |
| Vascular Pain | 3 | 186.48 | 59.68 | 582.65 | 185.92 | 545.99 | 183.98 | 70.92 | 7.52 | 5.85 |
| Cough | 3 | 0.63 | 0.2 | 1.96 | 0.63 | 0.64 | 0.63 | 0.25 | -0.66 | -2.33 |
| Pain In Extremity | 3 | 0.69 | 0.22 | 2.14 | 0.69 | 0.42 | 0.69 | 0.27 | -0.54 | -2.2 |
| Hepatic Enzyme Increased | 3 | 2.62 | 0.84 | 8.13 | 2.61 | 2.99 | 2.61 | 1.01 | 1.39 | -0.28 |
| Headache | 3 | 0.33 | 0.11 | 1.01 | 0.33 | 4.16 | 0.33 | 0.13 | -1.61 | -3.28 |
| Haemoptysis | 3 | 8 | 2.58 | 24.86 | 7.98 | 18.32 | 7.98 | 3.09 | 3 | 1.33 |
| Therapy Non-Responder | 3 | 3.13 | 1.01 | 9.71 | 3.12 | 4.33 | 3.12 | 1.21 | 1.64 | -0.03 |
| Contusion | 3 | 2.11 | 0.68 | 6.56 | 2.11 | 1.75 | 2.11 | 0.82 | 1.08 | -0.59 |
| Malaise | 3 | 0.5 | 0.16 | 1.55 | 0.5 | 1.52 | 0.5 | 0.19 | -1 | -2.67 |
| Lethargy | 3 | 4.31 | 1.39 | 13.38 | 4.3 | 7.59 | 4.29 | 1.66 | 2.1 | 0.43 |
| Thrombophlebitis | 3 | 81.44 | 26.15 | 253.6 | 81.2 | 236.54 | 80.83 | 31.24 | 6.34 | 4.66 |
| Cytopenia | 3 | 10.96 | 3.53 | 34.06 | 10.93 | 27.06 | 10.93 | 4.23 | 3.45 | 1.78 |
| Pulmonary Embolism | 3 | 2.83 | 0.91 | 8.8 | 2.83 | 3.55 | 2.83 | 1.1 | 1.5 | -0.17 |
| Infusion Site Vesicles | 3 | 316.01 | 100.72 | 991.46 | 315.06 | 922.62 | 309.52 | 118.9 | 8.27 | 6.59 |
| Lung Neoplasm Malignant | 3 | 2.46 | 0.79 | 7.64 | 2.45 | 2.59 | 2.45 | 0.95 | 1.3 | -0.37 |
| Therapy Change | 3 | 19.41 | 6.25 | 60.33 | 19.36 | 52.18 | 19.34 | 7.49 | 4.27 | 2.6 |
| Pleural Effusion | 3 | 3.8 | 1.22 | 11.8 | 3.79 | 6.16 | 3.79 | 1.47 | 1.92 | 0.25 |
| Lymphopenia | 3 | 11.52 | 3.71 | 35.81 | 11.49 | 28.73 | 11.49 | 4.45 | 3.52 | 1.85 |
| Hypoaesthesia | 3 | 1.45 | 0.47 | 4.49 | 1.44 | 0.41 | 1.44 | 0.56 | 0.53 | -1.14 |
| Cervical Vertebral Fracture | 3 | 57.11 | 18.35 | 177.71 | 56.94 | 164.36 | 56.76 | 21.96 | 5.83 | 4.16 |
| Hypokinesia | 2 | 8.43 | 2.11 | 33.78 | 8.42 | 13.07 | 8.41 | 2.63 | 3.07 | 1.4 |
| Rash | 2 | 0.28 | 0.07 | 1.14 | 0.29 | 3.61 | 0.29 | 0.09 | -1.81 | -3.48 |
| Swelling | 2 | 1.2 | 0.3 | 4.82 | 1.2 | 0.07 | 1.2 | 0.38 | 0.27 | -1.4 |
| Confusional State | 2 | 0.93 | 0.23 | 3.72 | 0.93 | 0.01 | 0.93 | 0.29 | -0.11 | -1.77 |
| Cholestasis | 2 | 7.14 | 1.78 | 28.58 | 7.12 | 10.53 | 7.12 | 2.23 | 2.83 | 1.16 |
| Hepatocellular Injury | 2 | 8.95 | 2.24 | 35.86 | 8.94 | 14.09 | 8.93 | 2.8 | 3.16 | 1.49 |
| Respiratory Tract Infection | 2 | 5.11 | 1.27 | 20.45 | 5.1 | 6.59 | 5.1 | 1.6 | 2.35 | 0.68 |
| Oedema Peripheral | 2 | 1.59 | 0.4 | 6.37 | 1.59 | 0.44 | 1.59 | 0.5 | 0.67 | -1 |
| Hypoacusis | 2 | 2.18 | 0.54 | 8.71 | 2.17 | 1.27 | 2.17 | 0.68 | 1.12 | -0.55 |
| Blood Creatinine Increased | 2 | 2.15 | 0.54 | 8.6 | 2.15 | 1.22 | 2.15 | 0.67 | 1.1 | -0.57 |
| Performance Status Decreased | 2 | 39.58 | 9.87 | 158.72 | 39.5 | 74.89 | 39.41 | 12.33 | 5.3 | 3.63 |
| Covid-19 | 2 | 0.27 | 0.07 | 1.07 | 0.27 | 4.02 | 0.27 | 0.08 | -1.9 | -3.57 |
| Ketoacidosis | 2 | 20.22 | 5.05 | 81.03 | 20.18 | 36.43 | 20.16 | 6.31 | 4.33 | 2.66 |
| Full Blood Count Abnormal | 2 | 2.86 | 0.71 | 11.43 | 2.85 | 2.41 | 2.85 | 0.89 | 1.51 | -0.16 |
| Neutropenic Sepsis | 2 | 19.08 | 4.76 | 76.45 | 19.04 | 34.16 | 19.02 | 5.95 | 4.25 | 2.58 |
| Therapy Cessation | 2 | 2.33 | 0.58 | 9.34 | 2.33 | 1.52 | 2.33 | 0.73 | 1.22 | -0.45 |
| Hepatic Lesion | 2 | 27.91 | 6.96 | 111.9 | 27.86 | 51.71 | 27.82 | 8.71 | 4.8 | 3.13 |
| Myelodysplastic Syndrome | 2 | 11.39 | 2.84 | 45.63 | 11.37 | 18.91 | 11.36 | 3.56 | 3.51 | 1.84 |

Supplementary Table 2：Disproportionality analysis of established signals for lurbinectedin compared to similar therapeutic drugs.

| PTs | Cases | ROR (95% CI) | PRR (χ^2^） | EBGM (EGBM_05_) | IC (IC_025_) |
| --- | --- | --- | --- | --- | --- |
| Death* | 22 | 15.19(9.59-24.06) | 12.71(240.67) | 12.71(8.65) | 3.67(1.98) |
| Disease Progression* | 11 | 51.81(27.91-96.19) | 47.38(500.26) | 47.37(28.23) | 5.57(3.88) |
| Platelet count decreased* | 4 | 8.82(6.95-50.96) | 18.26(65.35) | 18.25(7.93) | 4.19(2.50) |
| Thrombocytopenia* | 4 | 18.25(6.74-49.40) | 17.70(63.13) | 17.70(7.69) | 4.15(2.46) |
| Respiratory failure* | 3 | 20.00(6.36-62.88) | 19.55(52.87) | 19.55(7.50) | 4.29(2.60) |
| Neutrophil count decreased* | 3 | 38.88(12.37-122.22) | 37.98(108.06) | 37.97(14.56) | 5.25(3.56) |
| Neoplasm progression* | 3 | 41.49(13.20-130.43) | 40.53(115.71) | 40.52(15.54) | 5.34(3.66) |
| Extravasation* | 2 | 227.52(56.25-920.29) | 223.92(443.64) | 223.80(69.51) | 7.81(6.12) |
| Nausea | 2 | 1.24(0.31-5.01) | 1.24(0.09) | 1.24(0.38) | 0.31(-1.37) |
| White blood cell count decreased* | 2 | 9.12(2.25-36.86) | 8.99(14.22) | 8.99(2.79) | 3.17(1.48) |
| Hypoxia* | 2 | 28.74(7.11-116.21) | 28.30(52.69) | 28.30(8.79) | 4.82(3.14) |
| Dyspnoea | 2 | 1.73(0.43-6.98) | 1.72(0.60) | 1.72(0.53) | 0.78(-0.90) |
| Tumour lysis syndrome* | 2 | 120.20(29.72-486.09) | 118.30(232.59) | 118.27(36.74) | 6.89(5.20) |
| Administration site extravasation* | 2 | 1163.92(287.30-4715.30) | 1145.46(2280.46) | 1142.21(354.29) | 10.16(8.47) |
| Malignant neoplasm progression* | 2 | 10.20(2.52-41.25) | 10.06(16.33) | 10.05(3.12) | 3.33(1.64) |
| Lung neoplasm malignant* | 2 | 22.25(5.50-89.96) | 21.91(39.94) | 21.91(6.81) | 4.45(2.77) |
| Injection site extravasation* | 2 | 69.44(17.17-280.80) | 68.35(132.75) | 68.34(21.23) | 6.09(4.41) |
| Atrial fibrillation* | 2 | 10.02(2.48-40.51) | 9.88(15.98) | 9.88(3.07) | 3.30(1.62) |
| Injection site reaction* | 2 | 14.39(3.56-58.19) | 14.18(24.53) | 14.18(4.41) | 3.83(2.14) |
| Pneumonia | 1 | 1.44(0.20-10.28) | 1.43(0.13) | 1.43(0.28) | 0.52(-1.16) |

*Represents the simultaneous fulfillment of four algorithms

Supplementary Table 3：Clinical characteristics of potential new signals for lurbinectedin.

| Characteristics | Variable | N(%) |
| --- | --- | --- |
| Overall |  | 35(100%) |
| Gender | Male | 13 (37.1%) |
|  | Female | 13 (37.1%) |
|  | Unknown | 9 (25.7%) |
| Age(years) | 18-64 | 8 (22.9%) |
|  | 65-85 | 12 (34.3%) |
|  | Unknown | 15 (42.9%) |
| Report country | United States | 22 (62.8%) |
|  | France | 7 (20.0%) |
|  | Canada | 3 (8.6%) |
|  | Others | 3(8.6%) |
| Reporter's occupation | Consumer | 7 (20.0%) |
|  | Pharmacist | 7 (20.0%) |
|  | Physician | 19 (54.3%) |
|  | Others | 2 (5.7%) |

Supplementary Table 4:Concomitant medications and combined adverse event reports for potential new signals.

| Variable | Aki | Tumor lysis syndrome | Pneumonia | Concomitant medications |
| --- | --- | --- | --- | --- |
| Aki | - | 0 | 0 | 0 |
| Tumor lysis syndrome | 0 | - | 2 | 0 |
| Pneumonia | 0 | 2 | - | 0 |

Supplementary Table 5 ：Disproportionality analysis after removing confounding factors in lung tumor.

| PTs | Cases | ROR (95% CI) | PRR (χ^2^） | EBGM (EGBM_05_) | IC (IC_025_) |
| --- | --- | --- | --- | --- | --- |
| Death | 22 | 1.45(0.93-2.26) | 1.43(2.77) | 1.40(0.97) | 0.49(-1.18) |
| Neutropenia | 17 | 2.97(1.77-4.98) | 2.89(18.73) | 2.66(1.73) | 1.41(-0.27) |
| Disease Progression | 16 | 2.59(1.53-4.40) | 2.53(13.45) | 2.37(1.52) | 1.24(-0.44) |
| Nausea | 14 | 2.24(1.28-3.92) | 2.20(8.42) | 2.09(1.31) | 1.06(-0.62) |
| Thrombocytopenia | 12 | 2.75(1.50-5.07) | 2.71(11.54) | 2.51(1.51) | 1.32(-0.36) |
| Fatigue | 11 | 2.44(1.30-4.59) | 2.40(8.16) | 2.26(1.33) | 1.17(-0.51) |
| Sepsis | 9 | 3.51(1.72-7.16) | 3.46(13.56) | 3.11(1.71) | 1.64(-0.07) |
| Asthenia | 8 | 1.90(0.92-3.95) | 1.89(3.08) | 1.81(0.98) | 0.86(-0.83) |
| Febrile Neutropenia | 8 | 0.76(0.37-1.54) | 0.76(0.59) | 0.77(0.42) | -0.37(-2.05) |
| Pneumonia | 8 | 1.48(0.72-3.05) | 1.47(1.13) | 1.44(0.78) | 0.52(-1.16) |
| Constipation | 8 | 2.40(1.15-5.01) | 2.37(5.74) | 2.23(1.20) | 1.16(-0.54) |
| Anaemia | 8 | 0.86(0.42-1.76) | 0.86(0.17) | 0.87(0.48) | -0.20(-1.88) |
| Platelet Count Decreased | 7 | 1.40(0.65-3.03) | 1.40(0.75) | 1.37(0.72） | 0.46(-1.23) |
| Phlebitis* | 6 | 126.20(15.16-1050.59) | 124.46(105.05) | 18.64(3.16) | 4.22(2.26) |
| Infusion Related Reaction* | 6 | 7.87(3.07-20.22) | 7.78(25.84) | 5.93(2.69) | 2.57(0.79) |
| Acute Kidney Injury | 5 | 2.22(0.88-5.61) | 2.21(3.00) | 2.09(0.96) | 1.06(-0.65) |
| Dyspnoea | 5 | 0.74(0.30-1.81) | 0.74(0.44) | 0.75(0.35) | -0.41(-2.10) |
| Decreased Appetite | 5 | 1.10(0.45-2.73) | 1.10(0.05) | 1.10(0.52) | 0.14(-1.56) |
| Diarrhoea | 5 | 0.70(0.29-1.72) | 0.71(0.60) | 0.72(0.34) | -0.48(-2.17) |
| Malignant Neoplasm Progression | 5 | 1.06(0.43-2.61) | 1.06(0.02) | 1.06(0.50) | 0.08(-1.61) |

*Represents the simultaneous fulfillment of four algorithm
